# Power suppression in EEG after the onset of SAH is a significant marker of early brain injury in rat models

**DOI:** 10.1038/s41598-024-52527-0

**Published:** 2024-01-27

**Authors:** Yuji Takasugi, Tomohito Hishikawa, Tomohisa Shimizu, Satoshi Murai, Jun Haruma, Masafumi Hiramatsu, Koji Tokunaga, Yoshimasa Takeda, Kenji Sugiu, Hiroshi Morimatsu, Isao Date

**Affiliations:** 1 Department of Neurological Surgery, Okayama University Graduate School of Medicine, Dentistry and Pharmaceutical Sciences, Okayama, Japan; 2 Department of Neurological Surgery, Okayama University Faculty of Medicine, Dentistry and Pharmaceutical Sciences, Okayama, Japan; 3 Department of Neurosurgery, Okayama City Hospital, Okayama, Japan; 4 Department of Anesthesiology, Faculty of Medicine, Toho University, 6-11-1 Omori-nishi, Ota-ku, Tokyo, 143-8541 Japan; 5 Department of Anesthesiology and Resuscitology, Okayama University Faculty of Medicine, Dentistry and Pharmaceutical Sciences, Okayama, Japan

**Keywords:** Biophysics, Neuroscience, Physiology, Diseases, Medical research, Neurology, Signs and symptoms

## Abstract

We analyzed the correlation between the duration of electroencephalogram (EEG) recovery and histological outcome in rats in the acute stage of subarachnoid hemorrhage (SAH) to find a new predictor of the subsequent outcome. SAH was induced in eight rats by cisternal blood injection, and the duration of cortical depolarization was measured. EEG power spectrums were given by time frequency analysis, and histology was evaluated. The appropriate frequency band and recovery percentage of EEG (defined as EEG recovery time) to predict the neuronal damage were determined from 25 patterns (5 bands × 5 recovery rates) of receiver operating characteristic (ROC) curves. Probit regression curves were depicted to evaluate the relationships between neuronal injury and duration of depolarization and EEG recovery. The optimal values of the EEG band and the EEG recovery time to predict neuronal damage were 10–15 Hz and 40%, respectively (area under the curve [AUC]: 0.97). There was a close relationship between the percentage of damaged neurons and the duration of depolarization or EEG recovery time. These results suggest that EEG recovery time, under the above frequency band and recovery rate, may be a novel marker to predict the outcome after SAH.

## Introduction

The major pathophysiological factors contributing to poor outcome after subarachnoid hemorrhage (SAH) remain unexplained. Reversal of angiographic vasospasm after SAH does not lead to significant improvement in overall outcome^1,2^. Instead, early brain injury (EBI), which is brain damage occurring in the acute stage of SAH, has been receiving attention as one of the key factors affecting the prognosis after aneurysmal SAH^2,3^. It is reported that brain injury initiates within minutes after the initial bleed, and the injury came to called as EBI, which is the main cause of delayed ischemic neuronal deficits after SAH^10^. Sehba et al.^2^ reported that the EBI occur within 72 h after aneurysmal SAH (aSAH). There are some pathophysiological explanations to the relevance of EBI for the prognosis after aSAH. Sehba et al.^2^ summarized and reported that pathophysiological changes, such as increasing intracranial pressure, ionic derangement, electrophysiologic changes on EEG, molecular disturbance, are thought to cause the EBI. The precise physiological mechanisms and clinical markers of EBI remain unknown, however.

Loss of consciousness (LOC) is often seen in SAH patients in the emergency clinical situation, and the duration of LOC at the onset of SAH is strongly correlated with poor outcome^4–7^. EEG at the onset of LOC in head-up-tilt test demonstrated generalized slowing as a result of cerebral hypoperfusion, followed by generalized suppression during asystole and generalized slowing again on resumption of sinus rhythm (‘‘slow-flat-slow’’ pattern)^26^. Power change in continuously monitored electroencephalogram (EEG) may be another marker for the detection of delayed cerebral ischemia in patients with SAH^8,15,16^. An important finding has been reported that the duration of delayed suppression was correlated with the development of infarction and subsequent clinical outcomes^28^. Although an EEG power reduction lasting for several minutes after the induction of SAH is demonstrated in animal models^9,10^, the relationship between EEG recovery time immediately after the onset of SAH and prognosis is unknown. We hypothesized that EEG is useful and practical to predict the EBI^9^. We previously reported that ischemic depolarization, which indicates derangement of ion homeostasis and is seen in the brain of severe ischemic stroke^11^, also occurs at the onset of SAH, and the duration of ischemic depolarization is closely related to neuronal damage^12^. The purpose of this study was to analyze the correlations among membrane depolarization, changes in EEG, and histological outcome in rat models of SAH in the acute stage, and to determine the optimal combination of frequency band and duration of EEG recovery time to predict EBI after SAH.

## Methods

### Animals

All experiments were performed in accordance with the National Institutes of Health Guide for the Care and Use of Laboratory Animals and were approved by the Animal Research Control Committee of Okayama University Medical School. Male Sprague–Dawley (SD) rats (Charles River Laboratories Japan, Yokohama, Japan) in 9-week-old were used in this study. All experiments were reported in compliance with the ARRIVE (Animal Research: Reporting in Vivo Experiments) guidelines. The animals were fed ad libitum, and were deprived of food overnight prior to the experiments.

### General procedures

General anesthesia was induced with a mixture of 4% isoflurane (2-chloro-2-[difluoromethoxy]-1,1,1-trifluoro-ethane) in oxygen, and endo-tracheal intubation was performed on the animals. During the surgery, anesthesia was maintained by 1.5% isoflurane in 50% oxygen balanced with nitrogen, and artificial ventilation (SN-480–7; Shinano, Tokyo, Japan) was continuously maintained. The ventilation rate was adjusted to maintain blood gases within the physiological range.

A polyethylene catheter (SP 50, Natsume Seisakusho Co., Ltd., Tokyo, Japan) was placed in the right femoral artery for continuous monitoring of mean arterial blood pressure and blood sampling (i-STAT 300F, Abbott Point of Care, Princeton, NJ, USA). Another polyethylene catheter (SP 10, Natsume Seisakusho Co., Ltd., Tokyo, Japan) was connected carefully to a 30 gauge needle (Dentronics Needle, Handaya Co., Ltd., Tokyo, Japan), the tip of the needle was bent approximately 45 degrees so as not to damage the brain, and the other side of the polyethylene tube was connected to a 30 gauge cannula. After placing the rat in the stereotactic apparatus, the needle was inserted through the atlanto-occipital membrane and firmly secured in position with acrylic cement to fix the position of needle and seal the pinhole of the needle. This tube enabled blood injection with no direct contact with a rat.

**Figure 1:**
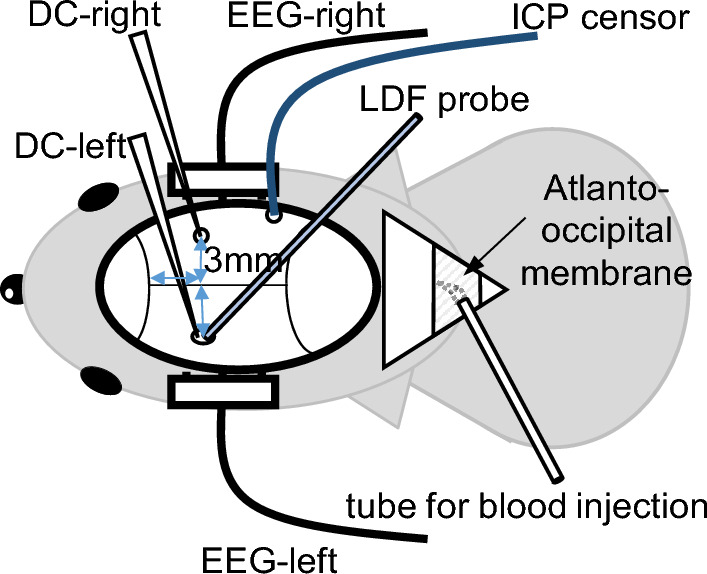
Schematic drawings of the preparation of a cannula for insertion through the atlanto-occipital membrane and of recording sites of DC potential, EEG, and ICP. A polyethylene catheter was connected carefully to a 30 gauge needle, the tip of the needle was bent approximately 45 degrees so as not to damage the brain, and the other side of the polyethylene tube was connected to a 30 gauge cannula. The positions of the DC electrodes were 3 mm posterior to the coronal suture and 3 mm lateral to the sagittal suture on both sides. To monitor the EEG, two pieces of needle electrodes were inserted in the bilateral temporal muscle. ICP was measured continuously using an ICP sensor inserted through a right temporal burr hole.

To monitor the extra-cellular potential, two borosilicate glass direct-current (DC) electrodes (tip diameter < 5 µm) were inserted into the brain surface at a depth of 750 µm through dural incisions made by a small burr hole. The positions of the electrodes were 3 mm posterior to the coronal suture and 3 mm lateral to the sagittal suture on both sides (Fig. 1). Duration of membrane depolarization was defined as the interval from the start of the sudden negative shift of extra-cellular potential to 80% recovery from maximal deflection^12^.

To monitor the EEG, two pieces of needle electrodes were inserted in bilateral temporal muscle adjacent to the DC electrodes in order to monitor the data nearby the DC electrodes, and finally histologic evaluation of this area. We used a set of gold-plated EEG electrodes to collect EEG data. The baseline recording was obtained before SAH induction. EEG raw data were analyzed using Origin 8.1 (OriginLab Corp., Northampton, MA). Power spectra were generated through time–frequency analysis, employing a minute-long epoch and a 5 Hz bandwidth spanning from 5 to 30 Hz via fast Fourier transform. Reference was made to our previous work on EEG power analysis utilizing Fourier transform for EEG decomposition^24^.

Intracranial pressure (ICP) was measured continuously using an ICP sensor (Codman® MicroSensor™, Codman & Shurtleff Inc., Raynham, MA) inserted through a right temporal burr hole.

Rectal temperature was monitored and maintained at 37.0 ± 0.5 °C using a heated water blanket. Epidural temperature was maintained at 37.0 ± 0.5 °C with a gentle flow of warmed saline (38.0 ± 0.5 °C) perfused over the skull surface.

All recorded variables were continuously digitized, displayed, and recorded by a computer using LabChart v7.3.7 and PowerLab data acquisition systems (ADInstruments, Dunedin, New Zealand).

### Induction of SAH

We adopted a single blood injection model of rat, as we previously reported, to induce the SAH^25^. Before the induction of SAH, the administered concentration of isoflurane was reduced to obtain baseline EEG activities. We check the EEG raw data and confirm the presence of awakening-like EEG pattern without burst suppression and isoelectricity, and recorded physiological data such as extra-cellular potential and EEG more than one minutes. SAH was initiated by the manually injection method. In brief, 0.2–0.3 ml autologous blood is collected from the right femoral artery and is slowly injected for 60 s into the cisterna magna through the tube positioned at the posterior region of the neck while monitoring the manually controlled ICP within the range of 40–80 mmHg.

After the induction of SAH, we observed the state of the rat to survive, and we collected the rat EEG dataset for 60 min. The EEG signals were collected at a sampling rate of 200 Hz. A total of approximately 60 min of EEG raw data were continuously obtained with minimal background noise, and the data were filtered using a low-cut filter at 5 Hz and a high-cut filter at 30 Hz. If significant deterioration of vital signs, such as declined mABP, abnormal respiratory or heart rates, was observed, we decided to stop the experiment.

### Histological evaluation

Seven days after the onset of SAH, all animals were anesthetized with 4% isoflurane. After inserting a cannula into the ascending aorta, each animal was perfused with heparinized physiologic saline (20 units/mL) and 6% paraformaldehyde in 0.1 mol/mL phosphate buffer (pH 7.4). The brain of each animal was removed, embedded in paraffin, and sectioned with a coronal section (thickness, 5 µm) at the levels of DC measurement. To identify the DC recording site, the location was recorded in a photograph for each rat at the time of electrode insertion. After perfusion-fixation, the DC recording site was marked using a 27 gauge needle and blue-black ink. All sections were stained with hematoxylin–eosin stain and photographed. The number of injured pyramidal neurons in the fifth layer of the brain cortex was counted by an observer who was blinded to the study. Pyramidal neurons exhibiting aggregated chromatin in the nucleus, shrinkage, or eosinophilic staining in the cytoplasm were considered injured^25^. The percentage of neuronal damage was calculated as the number of damaged neurons divided by the total number of neurons in the visual field × 100.

### Statistical analysis

Experimental data are shown as mean value ± standard deviation. All statistical comparisons were performed with the Student’s *t*-test. A level of *p* < 0.05 was considered to be significant in all statistical tests. In order to determine the appropriate frequency band of EEG among every 5 Hz band from 5 to 30 Hz and the appropriate EEG recovery time obtained from each rate of amplitude among 30%, 40%, 50%, 60%, and 70% recovery to predict the neuronal damage of cells within a field of view of microscope, 25 receiver operating characteristic (ROC) curve patterns in the combination of 5 frequency bands and 5 recovery rates were drawn with JMP® 11 (SAS Institute Inc., Cary, NC). Next, a probit regression curve, which expresses the probability of occurrence and is generally used to identify the median lethal dose in toxicology, was depicted by Origin 8.1 to evaluate the relationships between neuronal injury and total duration of membrane depolarization at the site of the DC electrode, and between neuronal injury and the EEG recovery time.

## Results

We used eight SD rats and all were fed appropriately during the experiments. The values of physiologic parameters obtained before SAH induction are shown in Table 1. We carefully checked the body weights of the rats in detail to ensure that the physiological data were consistent between rats. In all rats, the values of all parameters were maintained within normal limits before the induction of SAH. DC potential was successfully measured at a total of 16 DC electrode sites, and EEG was also successfully measured at 16 sites adjacent to the DC electrode.

**Table 1:** This is the physiological parameters in each series of experiments.

	Body weight (g)	pH	PaO2 (mmHg)	PaCO2 (mmHg)	BS (mg/dL)	Hb (g/dL)
n = 8	301 ± 12	7.417 ± 0.037	157 ± 39	41.6 ± 6.3	145 ± 41	12.6 ± 0.6

In all rats, mABP, blood gases (PaCO2, PaO2), pH, blood glucose (BS), and hemoglobin were within normal ranges before the induction of SAH.

PaO2, arterial oxygen pressure; PaCO2, partial pressure of carbon dioxide (arterial); BS, blood sugar; Hb, hemoglobin.

### Optimal measurement of EEG frequency band and recovery time to predict neuronal damage

**Figure 2:**
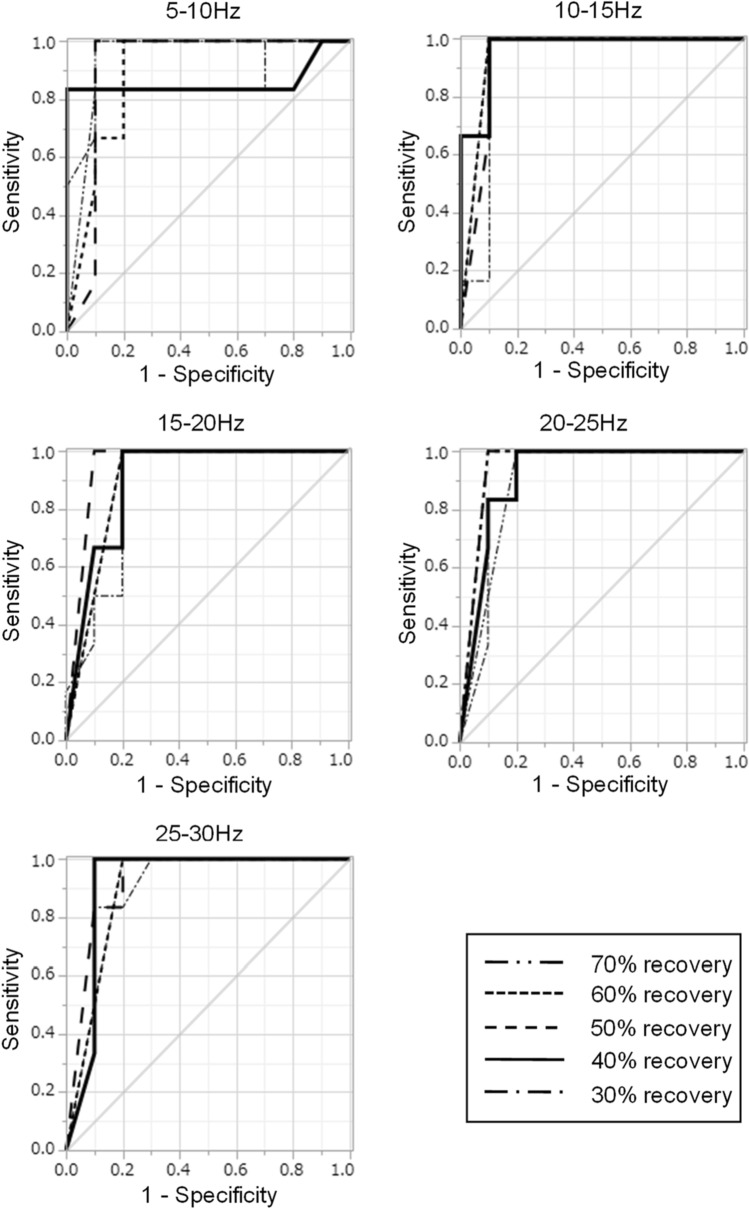
ROC analysis to determine the optimal EEG frequency band and recovery time to predict the presence of neuronal damage. To define the site with neuronal damage, we set the threshold of neuronal damage at 30% of cells within a field of view. A total of 25 patterns (5 frequency bands × 5 recovery rates) of graphs of ROC curves were given.

We assessed the relation between duration of EEG recovery time and histologic data. Histological evaluation was successfully performed in eight rats at the 16 sites around the DC electrode, and varied severity of neuronal damage was observed. To define a site with neuronal damage, we set the threshold of neuronal damage at 30% of cells within a microscope field of view. At six DC sites, neuronal damage was 30% or more whereas 10 DC sites exhibited less than 30% neuronal damage. The relative powers of common frequency bands (delta, theta, alpha, and beta waves) of EEG were 34.0%, 19.8%, 16.8%, and 29.3%, respectively. These values were comparable to the previous data, which reported percentages of 35.2%, 21.5%, 16.9%, and 26.4%, respectively. This data was extracted from a published graph^27^. The optimal frequency spectrum was analyzed for each frequency band (5–10 Hz, 10–15 Hz, 15–20 Hz, 20–25 Hz, and 25–30 Hz). The EEG recovery time in each frequency band was measured in five recovery percentages (30%, 40%, 50%, 60%, and 70%, respectively). From each frequency band and recovery percentage, 25 patterns in total (5 frequency bands × 5 recovery rates) of graphs of ROC curves for prediction of the presence of neuronal damage ≥ 30% were given (Fig. 2).

**Figure 3:**
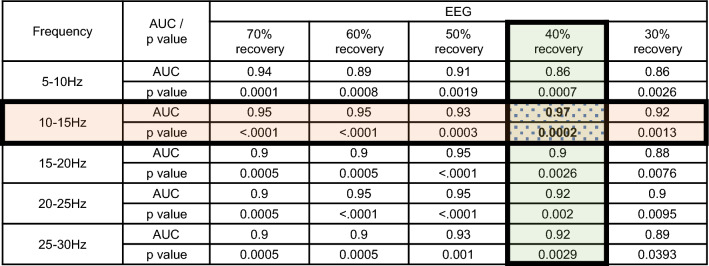
Based on the ROC curves in Fig. 2, we found that, for prediction of the presence of neuronal damage, the optimal frequency band of EEG and the duration from the beginning of EEG suppression to 40% recovery of power were 10–15 Hz and 40%, respectively (AUC: 0.97, *p* = 0.0002).

From these ROC curves, we calculated the area under the curve (AUC) and found that, to predict the presence or absence of neuronal damage at each site, the optimal frequency band of EEG and the EEG recovery time were 10–15 Hz and 40%, respectively (AUC: 0.97, *p* = 0.0002) (Fig. 3).

### Correlation among histologic outcome, DC potential, and EEG power analyzed data

**Figure 4:**
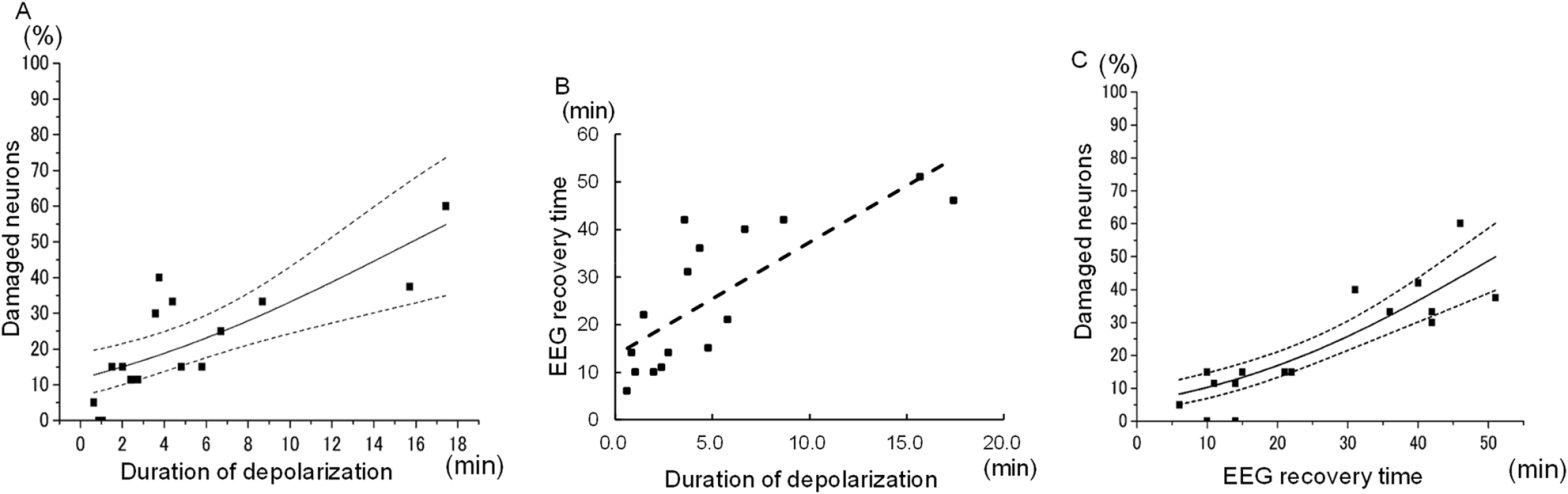
The logistic regression analysis to identify the correlations among the duration of depolarization, EEG recovery time, and percentage of neuronal damage. The logistic regression curve shows the close relation between duration of depolarization and histologic outcome at DC recording sites (probit curve, *r*^2^ = 0.796*, p* < 0.0001) (**A**). The duration of depolarization that induces ischemic cell changes in 50% of pyramidal neurons was estimated to be 15.9 min. By adopting the 10–15 Hz frequency band of EEG and the 40% recovery rate, we measured the EEG recovery time. The EEG recovery time and the duration of depolarization were closely correlated (r = 0.784, *p* = 0.0003) (**B**). The close relation between EEG recovery time and histologic outcome at DC recording sites is also shown (probit curve, *r*^2^ = 0.796, *p* < 0.0001) (**C**). The P50 (EEG), the EEG recovery time that induced ischemic cell changes in 50% of pyramidal neurons, was estimated to be 51.6 min.

As shown in Fig. 4A, the logistic regression curve shows the close relation between duration of depolarization and histologic outcome at DC recording sites (probit curve, *r*^2^ = 0.796, *p* < 0.0001). The P50 (DC), that is, the duration of depolarization that induces ischemic cell changes in 50% of pyramidal neurons, was estimated to be 15.9 min. Next, by adopting the 10–15 Hz frequency band of EEG and the 40% recovery rate based on the abovementioned findings, we measured the EEG recovery time and found that it was closely correlated with the duration of depolarization (r = 0.784, *p* = 0.0003) (Fig. 4B).

Figure 4C shows the logistic regression curve of the relation between EEG recovery time and histologic outcome, which showed a close relation between EEG recovery time and histologic outcome at DC recording sites (probit curve, *r*^2^ = 0.796, *p* < 0.0001). The P50 (EEG), that is, the EEG recovery time that induced ischemic cell changes in 50% of pyramidal neurons, was estimated (along with corresponding 95% confidence intervals) to be 51.6 min (49.8–52.9 min).

### Representative animal data

**Figure 5:**
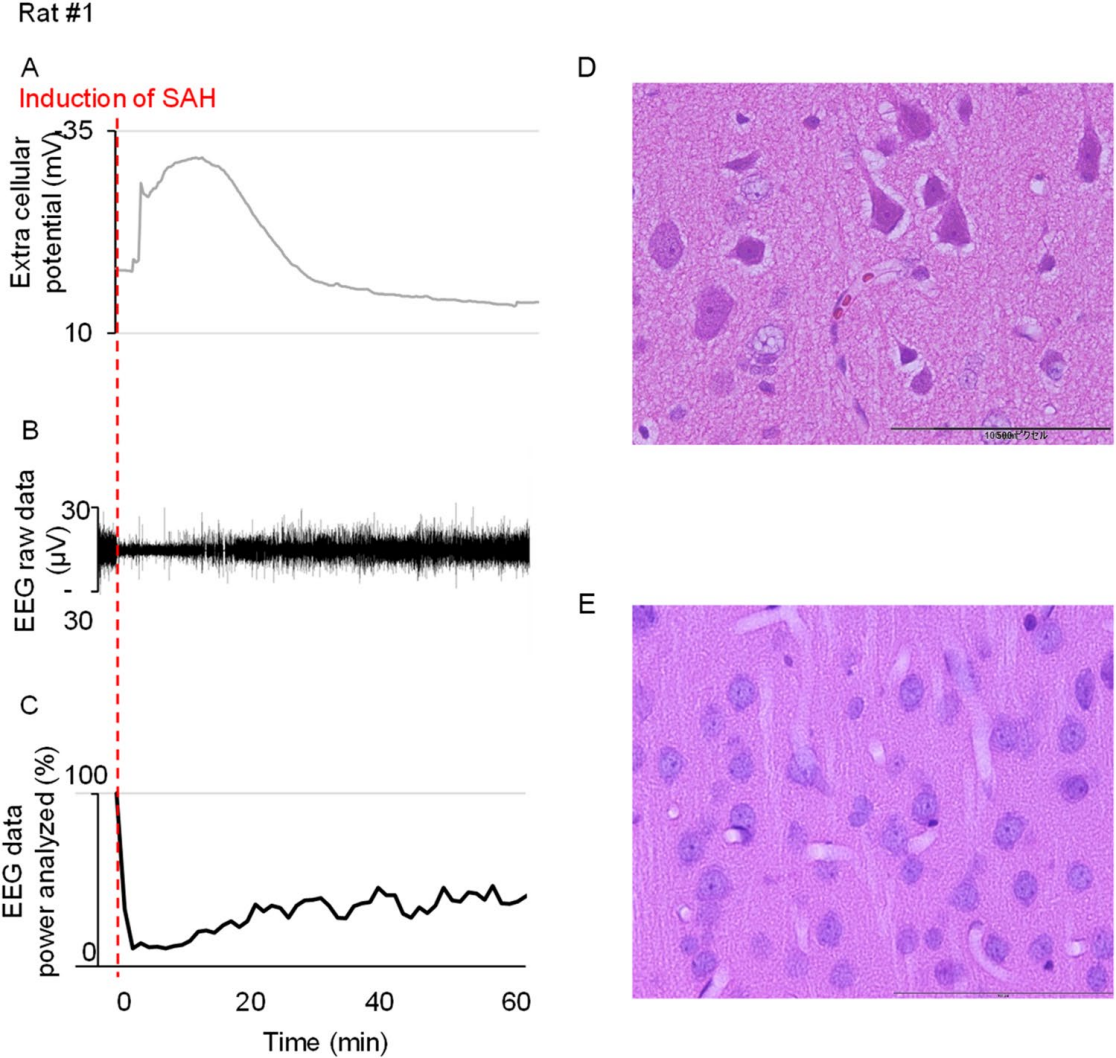
Representative data of DC, EEG, and histological outcome of Rat No.1. DC potential showed loss of membrane potential after the initiation of SAH and recovered to 80% of control levels in 17.4 min (**A**). EEG amplitudes were suppressed and flattened after the initiation of SAH, lasting several minutes and gradually recovering to some extent of the control level during 1 h observation periods (**B**, **C**). The duration necessary for 40% EEG power recovery of the 10–15 Hz frequency band was 46 min. Microscopic histological study of Rat No.1 revealed 60% neuronal damage in the field of view (**D**). Rat No.3 with short duration of depolarization and short EEG recovery time showed almost 0% neuronal damage (**E**).

Figure 5 shows the representative data of DC, EEG, and histological outcome of a rat. At DC electrode sites, DC potential showed loss of membrane potential after initiation of SAH and recovered to 80% of control levels in 17.4 min (Fig. 5A). EEG amplitudes were suppressed and flattened after initiation of SAH, lasting for several minutes and gradually recovering to some extent of the control level (Fig. 5B, C). The duration necessary for 40% EEG power recovery of the 10–15 Hz frequency band was 46 min. Microscopic histological study of this rat revealed 60% neuronal damage in the field of view.

## Discussion

The development of delayed ischemic neurological deficits (DINDs) is one of the major causes of poor outcomes in patients with SAH, but anti-vasospastic drugs failed to improve functional outcomes in clinical trials^1^. The pathological mechanisms are initiated in the very early stage (within 72 h) after the onset of SAH, and cause blood–brain barrier disruption, brain edema, inflammation, oxidative stress, and cell death, which are referred to as early brain damage (EBI)^9,13,14^. The details of the pathological mechanisms of EBI, however, remain unclarified. Recently, we successfully visualized cortical spreading depolarization in the acute phase of SAH in rats, though it was not associated with histological damage^12^. Elucidation of the mechanisms of EBI after SAH may lead to new therapeutic approaches to manage patients with SAH.

In our previous study in rat models with SAH induced by a stainless wire as a perforator for an intracranial artery, the longer depolarization persisted, the greater the rate of damaged neurons increased^12^. Van den Bergh et al.^17^ recorded DC potentials continuously following the induction of SAH in rat models, and reported that the brain lesion volume defined by MR diffusion-weighted images was smaller in the group in which the duration of depolarization was shortened by the administration of MgSO_4_ than in the control group showing the longer duration. In the present study, we estimated that the duration of depolarization that induced ischemic cell changes in 50% of pyramidal neurons was 15.9 min. In our aforementioned previous study, the duration of ischemic depolarization that caused histological neuronal damage in 50% was estimated to be 22.4 min^12^. In rat models with focal ischemia induced by occluding the middle cerebral and common carotid arteries, Higuchi et al.^11^ reported that the total time of depolarization causing 50% neuronal injury at the DC recording sites was estimated to be 18.2 min. In addition, they revealed that the sites showing depolarizations with short duration resulted in less neuronal injury whereas the sites showing recurrent depolarizations and eventually persistent depolarization resulted in infarction. The duration of depolarization causing 50% neuron injury was comparable among these studies, whether the mechanism of injury was ischemia or SAH, and in present study, we can predict the neuronal prognosis with single injection model of rat.

The present study demonstrated that the duration of depolarization was most closely related with the duration of EEG suppression by adopting a 40% recovery rate of the 10–15 Hz frequency band. Thus, it is reasonable to use EEG instead of DC measurement. This result is crucial in clinical situations because monitoring EEG is more common and easier in emergency suites than monitoring DC. The duration of EEG power suppression that caused damage to 50% of neurons was estimated to be 51.6 min, which was longer than the duration of depolarization until 80% recovery to cause the same damage. Since this is the first report in which EEG and DC are monitored simultaneously immediately after the induction of SAH and their correlation with early neuronal injury was analyzed, the relationships among the duration of EEG power reduction, recovery of DC potential, and neuronal cell damage at the very acute stage of SAH have not yet been elucidated elsewhere. In rat SAH models induced by vessel perforation, Bederson et al.^10^ reported that the mean intervals between the onset of SAH and the nadirs of cerebral perfusion pressure, cerebral blood flow, and EEG activity were 59, 189.5, and 289 s, respectively. Kamp et al.^9^ reported that injection of blood into the foramen magnum of mice instantly reduced total electrocorticographic power, reflecting the reduction of power in all frequency bands except for ripples, by an average of 65% and lasted for several minutes, but no obvious findings of EBI were demonstrated by neuropathological analysis. In animal ischemic models caused by vessel occlusion, the degree of neuronal cell injury was associated with the increasingly prolonged duration of electrocorticographic suppression^18,19^. Recently, Kentar et al., conducted research on electrocorticographic (ECoG) changes in a middle cerebral artery occlusion model of pigs. They observed spreading depolarizations passing through the brain cortex, causing severe disturbances across all frequency bands. The study advocated that ECoG could predict the progression of infarction^29^. Horst et al.^30^ conducted research in human SAH patients, and ECoG revealed a relationship among subarachnoid blood, delayed cerebral infarcts, and spreading depolarization. They suggested that therapeutic combination approaches should be further pursued. ECoG can be an effective means to predict brain damage and neurological outcomes. In patients in the subacute phase after SAH, Dreier et al.^20^ showed that the evolution of delayed infarcts was associated with prolonged electrocorticographic depression periods. Gollwitzer et al.^21^ reported that continuous EEG monitoring ranging from 2 to 12 days after SAH in 12 patients revealed a decrease of ≥ 40% in power persisting ≥ 5 h in the alpha band as the optimal cut-off to predict DCI. A prolonged duration of EEG depression can be considered as an indicator of energy depletion and impaired ion homeostasis in the animal and human brain^22,23^. While current methods for predicting delayed cerebral ischemia are known, they are invasive and complicated to perform in the emergency room before an aneurysm surgery^20–23^. In our present study, we found that EEG recovery time could be a predictor of EBI. For clinical application, monitoring the EEG during the acute stage of SAH with a portable instrument, such as the RD SedLine® EEG Sensor (Masimo Corporation, Irvine, California, USA), in the emergency suite is practical and easy. The EEG recovery time can predict the prognosis of SAH patients, aiding in the selection of surgical intervention and post-surgery medical treatment. This information is valuable for medical economy.

There are limitations in this study. It is not challenging to translate the results of this study into clinical feasibility for the detection of EBI. The correct frequency band of EEG to predict 50% neuron injury in human patients may not be the same as that for rats, so our findings should be interpreted cautiously. It is well known that certain anesthetics suppress EEG activity, reducing the cerebral metabolic rate, and prophylactic effects on ischemic neuronal damage. So, we need to know the deference between the experimental models and practical situations and cautiously translate the results of present study into practical human cases. And we did not measure the electrode’s impedance, but because we used a set of gold-plated EEG electrodes to collect EEG data, we assumed that electrode impedance would be similar in other rats. It may be an important limitation, but we calculated the relative EEG power of each animal, and it was comparable to a previous report^27^. Practically, the EEG of human patients with SAH cannot be monitored from the very beginning of onset. It may, however, be possible to at least predict the severity of EBI in patients, if the power reduction of EEG at certain frequency bands is recorded in the emergency room where patients are initially cared for immediately after the onset of SAH.

In conclusion, the suppression of EEG power defined by the appropriate frequency band and duration at the acute stage of SAH was closely correlated with the percentage of damaged neurons, and it may be a predictor of EBI in the very acute stage after SAH. The results of this study imply that the EEG data of patients in acute stage of SAH can be collected easily and timely in emergency suite, and the EEG recovery time will help to determine the treatment modality, and this novel discovery will contribute to make clear the mechanism of EBI as a new marker.

## Data Availability

The datasets used and/or analyzed during the current study available from the corresponding author on reasonable request.
